# Supplementary material for: Enhancing the forecast accuracy of the daily number of patients arrivals in emergency department by hybrid ARIMAX-ANN algorithm
Source: PLoS One. 2026 Apr 30;21(4):e0347866. doi: 10.1371/journal.pone.0347866 (PMC13132425; doi:10.1371/journal.pone.0347866)
Supplement: Attachment — Submitted filename: Response to reviewer.pdf [file pone.0347866.s003.pdf]

Dear Academic Editor of PLOS one,

Thank you for the opportunity to revise our work. We appreciate the valuable feedback from both reviewers and believe that addressing these comments will significantly improve the manuscript. Thank we highlighted in blue all the revisions. And here is the point by point response to the reviewer comments.

Thanks a lot and best regards,

Azadeh Saki (CA),

Associate Professor of Biostatistics, Department of Epidemiology and Biostatistics, School of Health, Mashhad University of Medical Sciences, Mashhad, Iran,
Mobile : +989163014565
E-mail : Sakia@mum.ac.ir; azadehsaki@yahoo.com

Reviewer #1:

This paper appears to be an attempt to propose an interesting model in the field of time series prediction, but it is not suitable for publication in its current state due to insufficient presentation of the problem definition, inadequate comparative experiments, limitations in performance evaluation, and lack of reproducibility.
In particular, it is essential to conduct a fair comparison with the latest time series models, clarify the hyperparameter optimisation process, introduce various performance metrics and statistical tests, and ensure the interpretability of variables. Additionally, the paper must present its applicability and research contribution in a more persuasive manner.
Therefore, this paper requires a major revision, and it is judged that it will only have publication value if the aforementioned issues are thoroughly addressed.

1. This paper aims to improve performance in specific prediction problems (time series-based), but it is unclear how it specifically fills the gap compared to existing research. It must be clarified whether it simply applies a new model or modifies an existing model, or whether it provides new insights in actual industrial/social applications. The motivation for ‘why this research is necessary’ is lacking. Thank you for your valuable comment. We add in the previous research gaps and necessity of our research in lines 90-96 and 115-135 of introduction.

2. The description of the data set's source, collection process, and preprocessing methods (missing value handling, outlier removal, normalisation, etc.) is insufficient. In

particular, in time series prediction research, the periodicity, seasonality, and trend characteristics of the data are important, and the results of exploratory analysis (ACF, PACF, time series decomposition, etc.) should be presented. Otherwise, it is difficult to judge the validity of the modelling. Thank you. There are no missing values in data set. And the outliers were not removed from data sets, because theses outliers are inherent behavior of the ED data set. The ACF and PACF show in Fig 5, and the periodicity and seasonality described in lines 553-563. Also there was no time trend (during years) to decomposition from time series before analysis.

3. The paper mentions hyperparameter settings during model training, but the rationale is insufficient. It should clearly state whether the default values were used or whether methods such as grid search or Bayesian optimisation were applied. The performance of time series prediction models is sensitive to parameters such as learning rate, window size, and hidden dimension, so failure to address these systematically weakens the credibility of the research. Thank you. We describe more in method section lines 281-285. "Extreme Learning Machine (ELM) for time series forecasting is a fast and efficient neural network-based approach that leverages a single-layer feedforward neural network (SLFN) structure. The key idea behind ELM is that the input weights and hidden layer biases are randomly assigned and remain fixed during training, while only the output weights are learned, typically by solving a linear system using matrix pseudo-inversion.

4. Only a few evaluation metrics such as RMSE or MAE were used, which are insufficient to adequately address model characteristics and problem definitions. It is necessary to present various metrics such as MAPE, SMAPE, and R^2 to verify prediction bias. In addition, statistical significance tests (e.g., Diebold-Mariano test) should be used to prove that the performance differences between models are meaningful. Thank you in advance for this valuable recommendation. We add all these indices in manuscript.

5. The paper focuses solely on improving model performance, but does not analyse which variables (features) contributed significantly to the prediction. In particular, time series prediction research needs to strengthen its explanatory power through variable importance (feature importance) and attention weight visualisation when considering practical application. Simply stating that 'the accuracy is high' limits the academic and practical contributions. Thank you. We add the results of ARIMAX in table1, and feature importance of ANN in Fig6. Also more details of variable importance in lines 572-577.

6. The reproducibility is low because the experimental code, parameter details, and hardware/software environment are not specified. In particular, ensuring reproducibility is important in the latest time series model research, but this paper overlooks this point. At the very least, pseudocode, data split method (train/valid/test ratio), and random seed settings should be provided. Thank you. We add all data and codes in supplementary files.

7. In the literature review, recent studies on deep learning models for time series prediction published in the last two to three years were not sufficiently cited. In particular, studies on Transformer-based prediction were omitted, which is a major flaw in clearly establishing the position of this study in academia. Thank you for your suggestion. Our literature review have two aspects: 1- hybride forecastying models 2- models for ED arrivals. We find and read this paper:
"Su, L., Zuo, X., Li, R., Wang, X., Zhao, H., & Huang, B. (2025). A systematic review for transformer-based long-term series forecasting. Artificial Intelligence Review, 58(3), 80."
So, we find that this models are not popular in ED arrival forecasting.

8. Lack of discussion on how the model presented in the paper can be applied in actual industries, policies, or services. It is difficult to judge the application value based solely on the result that 'prediction accuracy has improved.' Thank you. This concerns added in lines 652-672 of discussion.

Reviewer #2:
This manuscript addresses a highly relevant and practice-oriented issue: forecasting daily patient arrivals in emergency departments (EDs) by combining time series and machine learning techniques. The integration of calendar and meteorological variables with both ARIMAX and ANN, along with the development of two hybrid algorithms, is a valuable contribution to healthcare operations research. The comparative evaluation across multiple horizons (short, intermediate, and long term) further enhances the practical applicability of the findings. That said, several conceptual and methodological issues should be addressed to maximize the clarity and impact of the study. The manuscript would benefit from a more structured framing of the literature review, stronger justification for benchmark model selection, clearer reporting of ANN architecture and hyperparameter optimization, and deeper interpretation of horizon-specific performance differences. Additionally, highlighting the unique contributions of this study—beyond confirming the established role of calendar and meteorological factors—would strengthen its significance.

- The manuscript cites a large number of related studies in the introduction; however, these references are presented in a rather descriptive manner, without offering a coherent narrative. Summarizing the common implications and insights of prior research, and explicitly linking their limitations to the distinctive contributions of this study, would make the introduction more structured and persuasive.
- The study proposes two hybrid models and compares them with ARIMA and ANN. However, alternative hybrid models proposed in previous studies, such as the MA-filter based hybrid ARIMA–ANN algorithm (Babu et al., 2014) and the fuzzy time series algorithm (Jilani et al., 2019), were not included as benchmarks. The rationale for excluding these models should be clarified in more detail. Thank you for your valuable

comment. We add in the previous research gaps and necessity of our research in lines 90-96 and 115-135 of introduction. And revised the literature review accordingly.

- The manuscript mentions that 40 hidden nodes were used in the ANN model, but it is unclear whether the number of hidden layers was fixed at one. If a single hidden layer was adopted, was this the result of hyperparameter optimization or the authors' discretion? Furthermore, discussing how ANN performance changes with different configurations of hidden layers and nodes would strengthen the robustness of the modeling approach. Thank you. We describe more in method section lines 281-285. "Extreme Learning Machine (ELM) for time series forecasting is a fast and efficient neural network-based approach that leverages a single-layer feedforward neural network (SLFN) structure. The key idea behind ELM is that the input weights and hidden layer biases are randomly assigned and remain fixed during training, while only the output weights are learned, typically by solving a linear system using matrix pseudo-inversion.

- Figure 6 shows that the variable importance differs across ARIMAX and ANN, even when the same variables are used. An explanation of whether this difference stems from the contrast between linear and nonlinear approaches, or from other factors, would enhance the interpretability of the results. Thank you. We add the results of ARIMAX in table1, and feature importance of ANN in Fig6. Also more details of variable importance in lines 572-577.

- Typically, ANN analyses utilize a broad set of input variables to leverage its predictive capacity. In contrast, this study employed the Maximal Information Coefficient (MIC) approach for variable selection. Providing comparative evidence of model performance with and without the MIC approach would make the methodological contribution clearer. Thank you. The feature selection by MIC were done in ARIMAX not ANN. All input features included in ANN.

- It remains unclear how the dataset was divided into training, validation, and testing sets, and whether repeated experiments were conducted to ensure robustness. Since data splitting strategies and hyperparameter tuning can significantly affect model performance, Table 2 alone may not provide sufficient evidence to conclude that one model is superior to another. Thank you. In lines 172-177 we add details of test and train data partitioning: Model training utilized the initial 1008 days (equivalent to 144 weeks) of available data. To evaluate the robustness of algorithms and forecasting accuracies, the remaining 84 days (12 weeks) were held out for testing. This standardized train/test partition was maintained uniformly across the evaluation of all algorithms: Artificial Neural Network (ANN), ARIMAX, proposed Hybrid models, Long Short-Term Memory (LSTM), and Generalized Linear Model (GLM). Also, we addressed all these concerns in the manuscript.

- The results show that the best-performing model differs by horizon: Hybrid1 performs best in the short horizon, whereas Hybrid2 performs better in the intermediate horizon. The reasons for these differences should be discussed in more depth. Additionally, from a practitioner's perspective, guidance is needed on which model to use for short-, medium-, and long-term scheduling. If different models are to be applied depending on the time horizon, recommendations on how to integrate their outputs would be valuable.
Thank you. It is recommended that the Hybrid 2 is reasonable at all horizons.

- The finding that calendar and meteorological variables significantly influence patient arrivals is consistent with prior literature and may even be considered self-evident. The conclusion would be strengthened by emphasizing what is newly discovered in this study or highlighting its unique contributions beyond confirming established results.
Thank you. This concerns added in lines 652-672 of discussion, and in lines 729-735 of conclusion.
