# Supplementary material for: Enhancing the forecast accuracy of the daily number of patients arrivals in emergency department by hybrid ARIMAX-ANN algorithm
Source: PLoS One. 2026 Apr 30;21(4):e0347866. doi: 10.1371/journal.pone.0347866 (PMC13132425; doi:10.1371/journal.pone.0347866)
Supplement: Attachment — Submitted filename: Reviewer Response.docx [file pone.0347866.s005.docx]

**Manuscript ID: PONE-D-25-37594R1**

Dear Academic Editor of PLOS one,

Thank you for the opportunity to revise our work. We appreciate the valuable feedback from both reviewers and believe that addressing these comments will significantly improve the manuscript. We highlighted in blue all the revisions. And here is the point-by-point response to the reviewer comments.

Thanks a lot, and best regards,

Azadeh Saki (CA),

Associate Professor of Biostatistics, Department of Epidemiology and Biostatistics, School of Health, Mashhad University of Medical Sciences, Mashhad, Iran,

Mobile : +989163014565

E-mail : [Sakia@mum.ac.ir](mailto:Sakia@mum.ac.ir); azadehsaki@yahoo.com

Reviewer comments:

The manuscript proposes two hybrid prediction algorithms combining ARIMAX and ANN to forecast daily Emergency Department (ED) visits. Since the first round of review, the authors have made significant efforts to address the comments by enhancing data descriptions, expanding performance metrics, and adding the Diebold–Mariano (DM) test. The overall quality and reproducibility of the manuscript have noticeably improved.

However, from the perspective of a second-round review, the study still faces fundamental weaknesses regarding methodological novelty, the fairness of the comparative experiments, and the theoretical justification for the hybrid structures. While the manuscript is approaching the "technical soundness" criteria of PLOS ONE, further refinement is necessary before a final decision can be made. I recommend a Major Revision (borderline between major and minor).

1. Clarification of Research Contribution and Novelty

- The authors repeatedly emphasize "two novel hybrid algorithms" as a primary contribution. However, both Hybrid 1 (residual modeling) and Hybrid 2 (simple averaging) appear to be minor variations or standard combinations found in existing ARIMA–ANN literature. Specifically, Hybrid 2 is a basic form of ensemble forecasting, which makes the term "novel algorithm" feel somewhat overstated. The true contribution of this paper lies in its empirical application:
- Testing these models in a specific high-variability context (Mashhad, Iran).
- Incorporating exogenous variables through MIC-based selection.
- Demonstrating how these combinations stabilize predictions across intermediate horizons.
- Tone down the use of "novel algorithm" in the Introduction and Conclusion. Instead, explicitly frame the work as a context-specific empirical study that provides practical insights into ED management.

Thank you for this advance recommendation. We improve the manuscript accordingly in introduction lines 104-125, and discussion section lines: 653-668.

2. Fairness in Model Comparison (Experimental Design)

- While the authors added LSTM and GLM as requested, the setup for LSTM seems insufficient compared to the proposed models. The LSTM is presented with a single fixed setting (lookback=30, epoch=50, batch=30) without mention of hyperparameter tuning or repeated trials. In contrast, the ARIMAX and ANN (ELM) components underwent detailed pre-analysis, such as MIC and FDR. This creates an asymmetric level of optimization, making it difficult to determine whether the hybrid model's superiority over LSTM is due to architectural advantages or simply a lack of tuning for the LSTM baseline.
- At a minimum, clarify that the LSTM serves as an exploratory baseline. Ideally, provide a brief sensitivity analysis or explain the range of tuning used for the comparative models.

Thank you for your valuable comment. We improve the manuscript accordingly in introduction, lines: 139-143, and methods section, lines: 399-405.

3. Overstating the Role of MIC-based Feature Selection

- MIC-based feature selection was applied to ARIMAX and GLM, but ANN and LSTM reportedly received all variables. Despite this, the manuscript often attributes the hybrid model's success primarily to MIC. It remains unclear whether the performance gain in Hybrid 2 stems from:
  - The MIC-based selection process.
  - The bias-offsetting effect between ARIMAX and ANN.
  - The statistical stabilization inherent in simple averaging.
- Attribute the role of MIC specifically to "improving model interpretability and ARIMAX stability" rather than framing it as the sole driver of hybrid performance.

Thank you for your attention. We revise the manuscript accordingly in discussion section, lines: 678-693.
